# Dietary inflammatory index is associated with Vitamin D in CKD patients

**DOI:** 10.1007/s11255-023-03679-x

**Published:** 2023-06-28

**Authors:** Wenhui Wu, Xiurong Li, Jia Di, Hua Zhou, Hongyan Niu, Min Yang

**Affiliations:** Department of Nephrology, The Third Affiliated Hospital of Soochow University, No. 185 Juqian Road, Changzhou, 213003 Jiangsu Province China

**Keywords:** Dietary inflammatory index, 25-hydroxyvitamin D, Chronic kidney disease, National Health and Nutrition Examination Survey

## Abstract

**Objectives:**

Multiple observational studies have shown that low serum level of 25-hydroxyvitamin D (25(OH)D) in patients with chronic kidney disease (CKD) have been associated with a faster progression of kidney disease and a higher risk of all-cause mortality. We aim to assess the association between dietary inflammatory index (DII) with Vitamin D in adults with CKD.

**Method:**

The National Health and Nutrition Examination Survey appropriated participants from 2009 to 2018 were enrolled. The patients who were under the age of 18, pregnant, and having incomplete data were excluded. DII score were calculated based on a single 24-h dietary recall interview for each participant. Mutivariable regression analysis and subgroup analysis were utilized to determine the independent associations between vitamin D with DII in CKD patients.

**Results:**

In total, 4283 individuals were finally included. The results showed a negative association between DII scores and 25(OH)D with statistical significance (β =  – 1.83, 95% CI  – 2.31,  – 1.34, *P* < 0.001). In subgroup analysis stratified by gender, low eGFR, age and diabetes, the negative association between DII scores and 25(OH)D was still significant (all *P* for trend < 0.05). The results from interacion test indicated that the magnitude of the association was the same for the population with and without low eGFR (P for interacion = 0.464).

**Conclusion:**

Higher consumption of pro-inflammatory diet correlates negatively with the 25(OH)D level in CKD patients with and without low eGFR. Anti-inflammatory diet management may reduce the reduction of vitamin D in CKD patients.

## Introduction

Vitamin D is a group of fat-soluble steroids, which are essential for intestinal calcium absorption and for metabolic regulation of calcium and phosphates [1]. Vitamin D is either synthesized in the skin (vitamin D_3_) or ingested in the diet (vitamin D_2_) and is transported to the liver, where vitamin D 25-hydroxylase mediates D_2_/D_3_ to change into 25-hydroxyvitamin D (25(OH)D). 25(OH)D is the main storage form of vitamin D, which is a quantifiable form mostly used to determine vitamin D level in serum. The main physiologic function of vitamin D is to ensure adequate mineralization and bone growth [2]. The influence of vitamin D on the immune system is one of its another most important roles. Recent evidence show that vitamin D plays important role in metabolic syndrome (MetS), cardiovascular disease (CVD) [3], diabetes mellitus [4], and inflammation [5]. Prior studies have shown that 25(OH)D is negatively associated with markers of inflammation (interleukin [IL]-6, IL-10, high sensitivity C-reactive protein [hsCRP]) [6, 7]. Olsziwiec-Chlebna et al. reported that both the analogues of VitD (cholecalciferol and calcitriol) suppressed the proinflammatory cytokines (IL-17A and IL-23) in the airway of patients with Cystic Fibrosis [8]. However, an increasing number of authorities believed that chronic inflammation could lead to low 25(OH)D conversely [9]. Robert L. Modlin et al. found that cytokines, interferon-gamma and IL-4, stimulated conversion and catabolism of 25(OH)D respectively, resulting in low 25(OH)D in human monocytes [9, 10]. Another study showed that the extra-renal conversion of 25(OH)D by cytokines (TNF-α, IL-1, IL-2, IFN-γ, etc.) could result in depletion and low levels of 25(OH)D [10].

Chronic kidney disease (CKD) is a progressive loss of kidney function over time. The pathophysiological process of CKD is characterized by low-grade chronic inflammation [11]. Inflammation, together with coagulation disorders and neutrophil-endothelium interaction, are believed to play a role in the development of kidney injury, which may lead to chronically impaired function [12]. Compared to the healthy population, patients with CKD present more severe vitamin D deficiency and insufficiency [13]. Multiple observational studies have shown low levels of low 25(OH)D levels in patients with CKD and end-stage renal disease (ESRD) have been associated with a faster progression of kidney disease and a higher risk of all-cause mortality [14–16]. Many factors may account for low levels of 25(OH)D in CKD patients, including the loss of vitamin D binding protein in the urine [17], insufficient nutritonal intake, inadequate sun exposure and so on. In addition, inflammatory status might be an important factor contributing to the low low levels of 25(OH)D in CKD patients.

Diet play a central role in the regulation of chronic inflammation [18] and thus in kidney health. Anti-inflammation nutrients are associated with better kidney function [19, 20]. Conversely, pro-inflammation nutrients may be linked with worsening of kidney function [21]. Until 2009, there was no tool that could take into account the entire diet and determine its inflammatory potential. Researchers from University of South Carolina have developed a dietary tool called the Dietary Inflammatory Index (DII). DII, a literature-derived and population-based scoring system, was designed by assigning a score for each dietary parameter found to positively or negatively impact concentration of six specific inflammatory biomarkers: IL-1*β*, IL-4, IL-6, IL-10, TNF-*α* and CRP [22]. Forty-five pro and anti-inflammatory food parameters are included to calculate the DII score. A positive value for DII is assigned to an pro-inflammatory diet, and a negative value for DII is assigned to an anti-inflammatory diet. The higher total DII score indicate a more proinflammatory effect, and the lower total DII score suggest a more anti-inflammatory effect. The strength of the DII was that it evaluated the composite effects of multiple dietary components, rather than a single nutrient or individual food item. Recent studies have demonstrated that an increased DII not only affects the physical health of the patients such as cancer incidence [23, 24], all-cause and caner-specific mortality [25, 26] and respiratory conditions [27], but also has a significant effect on mental health [28]. Mazidi et al. has found that greater DII was associated with higher likelihood of chronic kidney disease [29]. However, the association between the dietary inflammatory potential and 25(OH)D has not been reported before. We hypothesized that increased intake of proinflammatory diets with increased levels of IL-1 β, IL-4, IL-6, IL-10, TNF- α and CRP was associated with decreased 25(OH)D levels in CKD patients.

In the present study, we aimed to assess the effect of DII on 25(OH)D in patients with CKD. We used the data from the National Health and Nutrition Examination Survey (NHANES), and estimated the negative relationship between DII and 25(OH)D levels. In addition, we further investigated this association in subgroups stratified by renal function, gender and age.

## Materials and methods

### Study population

We used data from the National Health and Nutrition Examination Survey (NHANES) database, which collect cross-sectional information such as demographic, socioeconomic, dietary, and medical data on American adults and children in two-year cycles through surveys, physical examination, and laboratory testing. The NHANES is administered by the National Center for Health Statistics (NCHS) which belongs to the U.S. Centers for Disease Control and Prevention (CDC). Trained interviewers and skilled personnel collected participants’ demographic, dietary information, clinical examination and laboratory examination. All NHANES data are publicly available at https://www.cdc.gov/nchs/nhanes/ and their protocols are approved by the institutional review board of the NCHS and all participants signed informed consent.

### Data collection

**Fig. 1:**
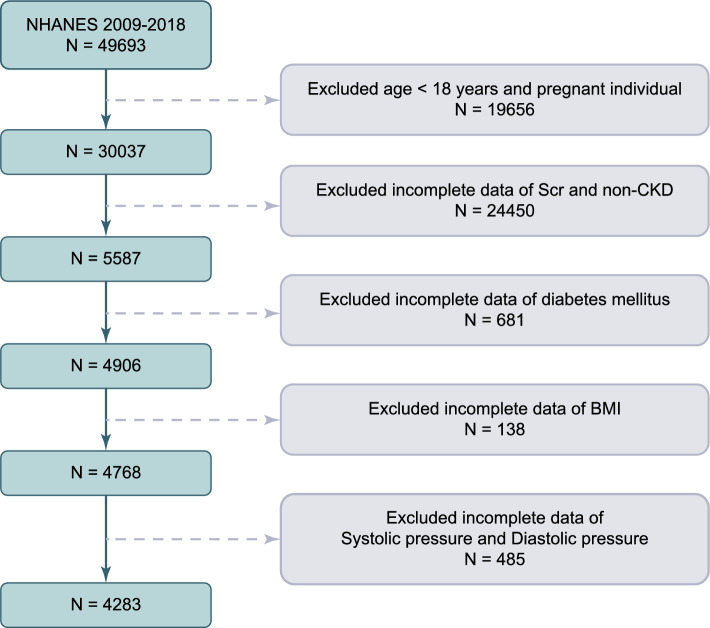
A flowchart of the sample selection from NHANES 2009–2018

For the present analysis, five survey cycles (i.e., 2009–2010, 2011–2012, 2013–2014, 2015–2016, 2017–2018) were combined to produce estimates with greater precision and smaller sampling error (*n* = 49,693). After excluding those population who were under the age of 18 (*n* = 19,341), pregnant (*n* = 315), missing the data of diabetes mellitus(*n* = 681), missing the serum creatinine data (used to determine eGFR) and non-CKD (*n* = 24,450), having incomplete data of Body Mass Index (BMI) (*n* = 138) and of Systolic pressure and Diastolic pressure (*n* = 485), the final analytical sample enrolled 4283 individuals from NHANES 2009–2018 (Fig. 1).

### Diagnostic criteria of CKD and diabetes mellitus

eGFR was calculated using the Chronic Kidney Disease Epidemiology Collaboration (CKD-EPI) equation[30] as flows: eGFR = 141 × min(Scr/*κ*, 1)^*α*^ × max(Scr/*κ*, 1)^−1*.*209^ × 0.993^*Age*^ × 1.018 (if female), in which Scr represents serum creatinine, *κ* represents the constant 0.7 for females and 0.9 for males, *α* represents  – 0.329 for females and – 0.411 for males, min is the minimum of Scr/*κ* or 1, and max indicates the maximum of Scr/*κ* or 1. According to the KDIGO guidelines: CKD stage 1, urinary albumin-to-creatinine ratio (ACR) ≥ 3 mg/mmol with eGFR ≥ 90 ml/min/1.73 m^2^; stage 2, ACR ≥ 3 mg/mmol with eGFR of 60–89 ml/min/1.73 m^2^; stage 3, eGFR of 30–59 ml/min/1.7 3m^2^ (with or without ACR ≥ 3 mg/mmol); stage 4, eGFR of 15–29 ml/min/1.73 m^2^, and stage 5, eGFR of < 15 ml/min/1.73 m^2^. The low-eGFR is defined as eGFR below 60 ml/min/1.73 m^2^.

A diagnosis of diabetes mellitus was considered as: (1). a fasting plasma glucose level ≥ 7.0 mmol/L, (2). hemoglobin A1c ≥ 6.5%, (3). prescribed hypoglycemic medications, (4) a history of diabetes.

### Exposure and outcome definitions

In the present study, the DII was designed as an exposure variable. The 24-h dietary data obtained via recall interviews were used to calculate the DII score for each appropriate individual. DII score was calculated as previously described [22]. A higher positive score value tended to indicate more pro-inflammatory property, and a more negative value indicated more anti-inflammatory [22]. A total of 26 food parameters were available in NHANES including anti-inflammatory food constituents (alcohol, *β* carotene, fibers, folic acid, magnesium, zinc, selenium, vitamin A, vitamin D, vitamin B-6, vitamin C, vitamin E, monounsaturated fatty acid, niacin, riboflavin, polyunsaturated fatty acid, caffeine, and thiamin), and pro-inflammatory food constituents (cholesterol, carbohydrates, energy, fats, iron, vitamin B-12, protein, and saturated fat). Previous studies have shown that the predictive ability of less than 30 dietary parameters was as accurate as more than 30 dietary parameters used to calculate DII score value [31, 32]. DII score was analyzed as a continuous variable, and was divided into tertiles for further analysis.

25(OH)D (nmol/l) was designed as an outcome variable and was measured by well trained technologists. 25(OH)D value was analyzed as a continuous variable.

### Other study variables

Other variables in this study consisted of age, gender, race, diabetes mellitus, systolic blood pressure (SBP, mmHg), diastolic blood pressure (DBP, mmHg), body mass index (BMI, kg/m^2^), serum glucose (mg/dl), serum creatinine (Scr, mg/dl), high-density lipoprotein (HDL, mmol/L), cholesterol (mmol/L), alkaline phosphatase (ALP, U/L), albumin (g/L), white blood cell (WBC, 1000 cells/*µ*L), vitamin D supplement (mg) (vitamin D_2_ and vitamin D_3_) and urinary albumin-to-creatinine ratio (ACR, mg/mmol). The measurement processes of the variables were publicly available at https://www.cdc.gov/nchs/nhanes/.

### Statistical analysis

Data are expressed as mean ± standard deviation and frequency or percentage for continuous and categorical variables respectively. Both one-way ANOVA analyses (for continuous variables) and Chi-square test (for categorical variables) were used to calculate the differences in three different DII groups. 25(OH)D values were analyzed as a continuous variable in all analyses. Multivariable linear regression model is utilized to determine the cross-sectional associations between 25(OH)D and DII in three different models. In model 1, no covariates were adjusted. Model 2 was adjusted for gender, age and race. Model 3 was adjusted for gender, age, race, diabetes mellitus, systolic blood pressure, diastolic blood pressure, BMI, serum glucose, eGFR, HDL, cholesterol, alkaline phosphatase, albumin, WBC, and ACR. The subgroup analysis was conducted by straitified multivariate regression analysis to further explore the relationship between DII and 25(OH)D in different population settings. In additon, an interaction item was added to test the heterogeneity of association between the subgroups. For all analyses, A *p-*value < 0.05 was considered statistically significant. All statistical analyses were performed using SPSS version 26(IBM).

## Result

### Baseline characteristics

The demographic characteristics and other covariates of the study subjects are described in Table 1. A total of 4283 participants were finally enrolled in this study. The average age was 62 ± 16 years old, with 2089 (48.8%) men and 2194 (51.2%) women. The DII score ranged from  – 4.95 to 4.69, with a median DII of 1.82 (0.19–3.15). The ranges of DII for tertiles 1–3 were  – 4.95 to 0.79, 0.79–2.71, and 2.71–4.69, respectively. The distribution of eligible individual and general characteristics by tertiles of the DII is shown in Table 1. For those different tertiles of DII, gender, race, serum creatinine, 25(OH)D, eGFR, low eGFR, systolic blood pressure, diabetes mellitus, and the proportions of different CKD stages were significantly different, but not for age, DBP, BMI, WBC count, HDL, albumin, ALP, serum calcium, serum phosphorus, cholesterol, serum glucose, TCG and vitamin D supplement. The proportion of males decreased from 57.6% to 48.8% (*P* < 0.001) across increasing tertiles of the DII. Across increasing DII tertiles, the proportion of non-Hispanic White (the largest ethnic group) and other Hispanic decreased; the proportion of non-Hispanic Black increased; the proportion of Mexican–American followed a reversed *U*-shape; and the proportion of remaining racial groups followed a *U*-shape (*P* < 0.001). Proportions of participants with diabetes mellitus, by DII tertiles, were 36.75% in the first tertile, 39.94% in the second tertile, and 42.21% in the top tertile (*P* = 0.011). The renal condition deteriorated across increasing tertiles of the DII. For instance, mean serum creatinie was 1.27 ± 0.98 mg/dl for the highest tertile group while 1.15 ± 0.81 mg/dl for the lowest tertile group (*P* = 0.002). Values (highest *v.* lowest DII tertiles) were 65.49 ± 29.37 *v.* 70.87 ± 27.49 ml/min/1.73 m^2^ for eGFR (*P* < 0.001); 53.1% *v.* 45.7% for prevalent low eGFR (*P* < 0.001). Across increasing DII tertiles, the proportion of CKD stage 1 and 2 decreased (*P* < 0.001); the proportion of stage CKD 3, 4 and 5 increased (*P* < 0.001). The level of SBP increased from 133.21 ± 21.46 mmHg in the lowest tertile to 135.41 ± 24.49 mmHg in the top tertile of the DII distribution (*P* = 0.032). Mean 25(OH)D was 71.26 ± 33.39 nmol/L, and the average 25(OH)D was 67.69 ± 32.6 nmol/L for the highest tertile group compared with 74.52 ± 32.97 nmol/L for the lowest tertile group (*P* < 0.001).

**Table 1:** Baseline characteristics of the participants according to different dietary inflammatory indexes(DII)

Clinica factors	Overall (*n* = 4283)	DII			*P* value
		T1	T2	T3	
	1.82 (0.19–3.15)	– 4.95 to 0.79 (*n* = 1428)	0.79–2.71 (*n* = 1427)	2.71–4.69 (*n* = 1428)	
Age ( years)	62.39 ± 16.98	62.10 ± 16.79	62.44 ± 16.88	62.63 ± 17.28	0.706
*Gender* (%)					< 0.001
Male	2089 (48.8%)	818 (57.6%)	677 (47.2%)	2089 (48.8%)	
Female	2194 (51.2%)	597 (42.4%)	760 (52.8%)	2194 (51.2%)	
*Race* (%)					< 0.001
Mexican American	501 (11.7%)	171 (12%)	177 (12.4%)	153 (10.7%)	
Other hispanic	347 (8.1%)	118 (8.4%)	121 (8.3%)	108 (7.6%)	
Non-hispanic white	1839 (42.9%)	662 (46.7%)	608 (42.5%)	569 (39.6%)	
Non-hispanic black	428 (27.3%)	309 (22%)	409 (28.3%)	450 (31.5%)	
Other race—including multi-racial	428 (10%)	155 (10.9%)	122 (8.5%)	151 (10.6%)	
Serum creatinine (mg/dL)	1.21 ± 0.92	1.15 ± 0.81	1.21 ± 0.96	1.27 ± 0.98	0.002
ACR (mg/mmol)	22.74 ± 88.62	24.21 ± 106.88	20.97 ± 80.37	22.05 ± 75.39	0.746
25(OH)D, serum (nmol/L)	71.26 ± 33.39	74.52 ± 32.97	71.57 ± 34.24	67.69 ± 32.6	< 0.001
25(OH)D_2_ (nmol / L)	6.16 ± 17.4	5.5 ± 15.35	6.24 ± 17.5	6.73 ± 19.15	0.058
25(OH)D_3_ (nmol / L)	65.14 ± 33.2	69.07 ± 33.03	65.37 ± 34.24	60.99 ± 31.8	< 0.001
eGFR (ml/min/1.73 m^2^)	68.16 ± 28.59	70.87 ± 27.49	68.1 ± 28.66	65.49 ± 29.37	< 0.001
*Low eGFR*					< 0.001
No	2015 (47%)	54.3%	49.4%	46.9%	
Yes	2268 (53%)	45.7%	50.6%	53.1%	
*CKD*					< 0.001
Stage 1	1067(24.9%)	26.4%	24.4%	23.9%	
Stage 2	948(22.1%)	24.6%	22.1%	19.8%	
Stage 3	2023(47.2%)	45.7%	47.5%	48.4%	
Stage 4	162(3.8%)	2.2%	4.2%	5.0%	
Stage 5	83(1.9%)	1.1%	1.8%	2.9%	
Body mass index (kg/m^2^)	41.98 ± 143.62	42.11 ± 145.12	42.95 ± 142.29	40.9 ± 143.52	0.929
Systolic blood pressure (mmHg)	134.41 ± 22.66	133.21 ± 21.46	134.61 ± 21.87	135.41 ± 24.49	0.032
Diastolic blood pressure (mmHg)	69.25 ± 15.78	69.71 ± 15.51	69.11 ± 15.65	68.92 ± 16.17	0.376
*Diabetes *(%)					0.011
No	2585 (60.4%)	895 (63.25%)	863 (60.06%)	827 (57.79%)	
Yes	1698 (39.6%)	520 (36.75%)	574 (39.94%)	604 (42.21%)	
HDL(mmol / L)	1.36 ± 0.44	1.36 ± 0.44	1.36 ± 0.45	1.35 ± 0.43	0.559
Calcium, serum (mmol/L)	2.35 ± 0.13	2.34 ± 0.15	2.35 ± 0.13	2.35 ± 0.1	0.054
Phosphorus, serum (mmol/L)	1.2 ± 0.2	1.21 ± 0.21	1.2 ± 0.18	1.2 ± 0.21	0.713
Vitamin D supplement (mg)	46.7 ± 142.33	44.51 ± 131.85	48.45 ± 156.37	47.85 ± 137.51	0.887

*T1* the first tertile, *T2* the second tertile, *T3* the top tertile, *eGFR* estimated glomerular filtration rate, *CKD* chronic kidney disease, *ACR* albumin-to-creatinine ratio, urinary, *HDL* high-density lipoprotein, *Low eGFR* eGFR < 60 ml/min/1.73 m^2^. CKD stage 1: eGFR ≥ 90 ml/min/1.73 m^2^ and ACR ≥ 3 mg/mmol; stage 2: 60 ml/min/1.73 m^2^ ≤ eGFR < 90 ml/min/1.73 m^2^ and ACR ≥ 3 mg/mmol; stage 3: 30 ml/min/1.73 m^2^ ≤ eGFR < 60 ml/min/1.73m^2^; stage4: 15 ml/min/1.73 m^2^ ≤ eGFR < 30 ml/min/1.73 m^2^; stage 5: eGFR < 15 ml/min/1.73 m^2^

Mean levels of 25(OH)D based on different conditions were shown in Table 2. The participants with low eGFR, with non diabetes mellitus, female and over 60 years of age tended to have higher 25(OH)D levels compared with their counterparts. In the low eGFR group, mean 25(OH)D was 83.14 ± 35.56 nmol/L for the lowest tertile group while 75.41 ± 34.65 nmol/L for the highest tertile group (*P* < 0.001). Mean 25(OH)D was 66.24 ± 28.89 nmol/L for the lowest tertile group while 57.76 ± 26.69 nmol/L for the highest tertile group (*P* < 0.001) in the non-low eGFR group. We also calculated the mean 25(OH)D level according to different stages of CKD. The 25(OH)D level in the patients with CKD, stages 1 to 5, was 57.17 ± 24.49, 67.80 ± 30.18, 79.83 ± 35.46, 78.59 ± 38.56, 68.57 ± 36.38 nmol/L. In the stage 1–5 group, 25(OH)D levels (highest *v.* lowest DII tertiles) were 54.69 ± 25.05 *v.* 60.99 ± 24.68 nmol/L (*P* = 0.001) for stage 1; 61.48 ± 28.14 *v.* 71.87 ± 30.0 nmol/L (*P* < 0.001) for stage 2; and 75.44 ± 33.87 *v.* 83.31 ± 35.45 nmol/L (*P* < 0.001) for stage 3; 80.94 ± 40.76 *v.* 86.94 ± 40.74 nmol/L (*P* = 0.469) for stage 4; 65.46 ± 34.88 *v.* 67.98 ± 26.05 nmol/L (*P* = 0.82) for stage 5. In the female group, mean 25(OH)D was 78.54 ± 34.41 nmol/L for the lowest tertile group while 67.76 ± 33.14 nmol/L for the highest tertile group (*P* < 0.001). In elderly group (over 60 years of age), average 25(OH)D was 81.1 ± 33.81 nmol/L for the lowest tertile group while 74.09 ± 32.92 nmol/L for the highest tertile group (*P* < 0.001). In non-diabetes group, average 25(OH)D was 77.2 ± 32.76 nmol/L for the lowest tertile group while 67.93 ± 32.98 nmol/L for the highest tertile group (*P* < 0.001); however there was no similar trend in the diabetes group (*P* = 0.159).

**Table 2:** Mean levels of 25(OH)D based on different conditions

	Overall	T1	T2	T3	*P* for trend
25(OH)D, serum (nmol/L)					
*Gender*					
Male	69.49 ± 31.27	71.56 ± 31.56	68.65 ± 30.26	67.58 ± 31.86	0.043
Female	72.94 ± 35.21	78.54 ± 34.41	74.17 ± 37.26	67.76 ± 33.14	< 0.001
*Low eGFR*					
No	62.17 ± 27.82	66.24 ± 28.89	61.85 ± 28.16	57.76 ± 26.69	< 0.001
Yes	79.33 ± 35.77	83.14 ± 35.56	79.96 ± 36.73	75.41 ± 34.65	< 0.001
*CKD*					
Stage 1	57.17 ± 24.49	60.99 ± 24.68	55.46 ± 23.26	54.69 ± 25.05	0.001
Stage 2	67.80 ± 30.18	71.87 ± 30.0	68.94 ± 31.29	61.48 ± 28.14	< 0.001
Stage 3	79.83 ± 35.46	83.31 ± 35.45	80.94 ± 36.62	75.44 ± 33.87	< 0.001
Stage 4	78.59 ± 38.56	86.94 ± 40.74	71.5 ± 33.88	80.94 ± 40.76	0.469
Stage 5	68.57 ± 36.38	67.98 ± 26.05	73.92 ± 43.82	65.46 ± 34.88	0.82
*Age*					
< 60 years	59.69 ± 28.01	63.88 ± 28.55	58.69 ± 27.46	55.76 ± 28.42	< 0.001
> 60 years	78.07 ± 34.07	81.1 ± 33.81	78.93 ± 35.55	74.09 ± 32.92	< 0.001
*Diabetes*					
No	72.66 ± 33.05	77.2 ± 32.76	72.47 ± 32.81	67.93 ± 32.98	< 0.001
Yes	69.13 ± 33.79	70.27 ± 32.89	69.87 ± 36.17	67.43 ± 32.15	0.159

*T1* the first tertile, *T2* the second tertile, *T3* the top tertile, *CKD* chronic kidney disease, Low eGFR: eGFR < 60 ml/min/1.73 m^2^. CKD stage 1: eGFR ≥ 90 ml/min/1.73 m^2^ and ACR ≥ 3 mg/mmol; stage 2: 60 ml/min/1.73 m^2^ ≤ eGFR < 90 ml/min/1.73 m^2^ and ACR ≥ 3 mg/mmol; stage 3: 30 ml/min/1.73 m^2^ ≤ eGFR < 60 ml/min/1.73 m^2^; stage 4: 15 ml/min/1.73 m^2^ ≤ eGFR < 30 ml/min/1.73 m^2^; stage 5: eGFR < 15 ml/min/1.73 m^2^

### The association between DII and 25(OH)D

Multivariable linear regression analysis was used to estimate the association of DII with 25(OH)D in three different models (Table 3). The results showed a negative association between DII scores and 25(OH)D with statistical significance (Model 1, β =  – 1.41, 95% CI  – 1.92,  – 0.90, *P* < 0.001; Model 2, β =  – 1.72, 95% CI  – 2.21,  – 2.23, *P* < 0.001; Model 3, β =  – 1.83, 95% CI  – 2.31,  – 1.34, *P* < 0.001). According to the fully adjusted model (model 3), 25(OH)D decreased by 1.83 nmol/L per one unit increase in the DII, which suggested that higher DII scores were associated with a lower 25(OH)D level. And this association remained statistically significant after DII was classified as tertiles. The fully adjusted effect size (Tertile 1 as reference) was  – 4.45 (95% CI  – 6.75,  – 2.14, *P* < 0.001) for Tertile 2,  – 8.96 (95% CI  – 11.28,  – 6.63, *P* < 0.001) for Tertile 3.

**Table 3:** Association between DII and 25(OH)D

	Β (95% CI) *p* value		
	Model 1	Model 2	Model 3
25(OH)D, serum (nmol/L)			
DII (continuous)	– 1.41 (– 1.92, – 0.90) *P* < 0.001	– 1.72 (– 2.21, – 2.23) *P* < 0.001	– 1.83 (– 2.31, – 1.34) *P* < 0.001
*DII categories*			
T1	0 (Ref)	0 (Ref)	0 (Ref)
T2	– 3.22 (– 5.66, – 0.78) *P* = 0.010	– 4.13 (6.46, – 1.79) *P* = 0.001	– 4.45 (– 6.75, – 2.14) *P* < 0.001
T3	– 6.94 (– 9.38,– 4.49) *P* < 0.001	– 8.41 (– 10.75,– 6.06) *P* < 0.001	– 8.96 (– 11.28, – 6.63) *P* < 0.001

Insensitivity analysis—dietary inflammatory index was converted from a continuous variable to a categorical variable (tertiles). 95%CI, 95% confidence interval; β: effect sizes

Model 1: no covariates were adjusted

Model 2: adjusted for gender, age, and race

Model 3: adjusted fo for age, gender, race, body mass index, diabetes mellites, systolic blood pressure, diastolic blood pressure, white blood cell, high-density lipoprotein, estimated glomerular filtration rate; albumin-to-creatinine ratio, urinary; alkaline phosphatase; albumin; serum glucose

### Subgroup analysis

We conducted the subgroup analysis stratified by gender, low eGFR, age and diabetes to further evaluate the association of DII with 25(OH)D level in different population settings through stratified multivariate regression analysis and test the interactions (Table 4). In both subgroups, the negative association between DII scores and 25(OH)D was still significant (all *P* for trend < 0.05), suggesting that the correlation between DII and 25(OH)D level was similar in the population with different gender, low eGFR, age, and diabetes status. Interaction test was performed to evaluate if there was any significant dependence of the effect modifier on the association; *P* for interaction > 0.05 means no significant dependence. The test for interacion were significant for gender (*P* for interacion = 0.001), age (*P* for interacion < 0.003), diabetes (*P* for interacion < 0.001), indicating significant dependence on gender, age, and diabetes status. However, we did not find any significant dependence on the low eGFR (*P* for interacion = 0.464), indicating that the magnitude of the association was the same for the population with/without low eGFR.

**Table 4:** Subgroup analysis stratified by different variables

DII	Β (95% CI), *P* for trend	*P* for interacion
*Gender*		
Male	– 3.23 (– 4.79, – 1.66) *P* < 0.001	0.001
Female	– 5.66 (– 7.38, – 3.94) *P* < 0.001	
*Low eGFR*		
No	– 4.46 (– 5.90, – 3.01) *P* < 0.001	0.464
Yes	– 4.18 (– 5.96, – 2.41) *P* < 0.001	
*Age*		
< 60 years	– 4.84 (– 6.53, – 3.14) *P* < 0.001	0.003
> 60 years	– 4.37 (– 5.91, – 2.82) *P* < 0.001	
Diabetes		
No	– 5.38 (– 6.83, – 3.93) *P* < 0.001	< 0.001
Yes	– 2.92 (– 4.84, – 0.99) *P* = 0.003	

Subgroup analysis stratified by different variables

The results show that the subgroup analysis was adjusted for all presented covariates of Model 3 except the effect modifier

eGFR: estimated glomerular filtration rate

β: effect sizes

95% CI: 95% confidence interval

### The correlation between25(OH)D and some food constituents

The association between 25(OH)D and some pro-inflammatory food constituents, including cholesterol, protein, total saturated fatty acids, total monounsaturated fatty acids and total polyunsaturated fatty acids, were evaluated using Pearson’s correlation analysis, as presented in Table 5. Protein, total saturated fatty acids, and total monounsaturated fatty acids, but not Cholesterol and total polyunsaturated fatty acids, were significantly and negatively associated with 25(OH)D. None of these five components was positively associated with 25(OH)D. However, a positive association was found between Vitamin D supplement and 25(OH)D (*r* = 0.135, *P* < 0.001).

**Table 5:** The correlation between 25(OH)D and some food onstituents

	25 (OH)D	
	Pearson’s coefficient	*P*
Cholesterol (mg)	– 0.028	0.071
Protein (g)	– 0.036	0.018
Total saturated fatty acids (g)	– 0.032	0.036
Total monounsaturated fatty acids (mg)	– 0.033	0.032
Total polyunsaturated fatty acids (mg)	– 0.009	0.542
Vitamin D supplement (mg)	0.135	< 0.001

## Discussion

This cross-sectional analysis documented the association between DII and 25(OH)D in patients with CKD. Our results demonstrated that higher consumption of pro-inflammatory diet leaded to lower 25(OH)D in CKD patients, even after adjustment for a range of extraneous factors. Moreover, we found that this negative association was remained in subgroup analysis stratified by low eGFR, gender, age, and diabetes, suggesting this relationship could be applicable to population with different condition. What's particularly interesting was that pro-inflammatory food constituents (such as protein, total saturated fatty acids, and total monounsaturated fatty acids, which were the main sources of 25(OH)D), were negatively associated with 25(OH)D.

It has been well documented that Vitamin D played a major role in bone metabolism. Over the past years, considerable pieces of evidence have demonstrated its effects on inflammation and immunity. Cherrie MPC et al. found an atopic dependent trend in the association between 25(OH)D levels and asthma [33]. Various studies have emphasized the association between low level of vitamin D with increased risk of respiratory disease symptoms [33, 34]. A cross-sectional study proposed that lower VitD levels might cause severity and more complications in treatment in asthmatic adults [35]. Rabih Halwani et al. revealed that VitD plays immunomodulatory role during COVID – 19 infection [36]. Studies have found that 25(OH)D is negatively associated with markers of inflammation (interleukin [IL]-6, and high sensitivity C-reactive protein (hsCRP) in Autism Spectrum Disorders [6, 7]. Vitamin D actions on inflammatory mechanisms depends on its biologically active form, 1,25(OH)2D. Generally, 1,25(OH)2D enhance the innate immune system and inhibit the adaptive immune system [33, 37]. As for innate immune system, 1,25(OH)2D binds to the vitamin D receptor (VDR), which is present in most immune cell types particularly in antigen-presenting cells (APCs) (monocytes, macrophages and dendritic cells) [38], and activates the VDR to express antimicrobial peptides (AMPs) such as cathelicidin and beta defensins to attack pathogens [39, 40]. For adaptive immune responses, 1,25(OH)2D binds to the VDR and modulates the balance of T-helper subsets by inhibiting Th1 and Th17 effector cells, and enhancing the development of Treg cells. 1,25(OH)2D suppressed the release of pro-inflammatory cytokines (e.g., IL-2, IL-6, IL-12, INFr, TNFa, etc.) from both innate and adaptive immune response [41].

Extensive research has been done to estimate the effect of inflammation on the 25 (OH) D level. In the present study, we concluded an inverse association between the pro-inflammation nutrients and 25 (OH) D level in patients with CKD. This cannot be fully explained by the above-mentioned mechanisms for dietary parameter as a invariable factor. Opposing reasoning can be used to explain this contradiction. One explanation reasons is that chronic inflammation can result in low level of 25(OH)D. Kelly Fincher et al. considered that after nucleated cells parasitized by intracellular bacteria, extra-renal production of 1,25 (OH)2D increased, and 25(OH)D decreased due to rapid conversion to 1,25 (OH) 2D for CYP27B1 activation [9]. Multiple mechanisms are thought to be involved in the pathogenesis: a. inflammatory cytokines (e.g., TNF-α, IL-1, IL-2 and IFN-γ) activates CYP27B1, an enzyme that converts 25(OH)D into its active form 1,25(OH)2D [42], which is expressed in most immune cell types such as macrophages [9, 10]; b. elevated 1,25(OH)2D binds to the PXR (pregnane X receptor) and inhibits conversion of Vitamin D_3_ to 25(OH)D [43]; c. excess 1,25(OH)2D inhibits the hepatic synthesis of 25(OH)D [44]. The hypothesis was confirmed by Waldronn et al. who found a reduction of the serum 25(OH)D following an acute inflammatory insult (i.e., orthopedic surgery) [45]. The DII was designed based on the impaction of dietary parameter on the inflammatory biomarkers (IL-1 β,  IL-4, IL-6, IL-10, TNF- α and CRP), which might stimulate the activation of CYP27B1.

Regarding the negative association between DII scores and the 25(OH)D level, we observed significant dependence on gender (*P* for interation = 0.001), age (*P* for interation = 0.003) and diabetes status (*P* for interation < 0.001), but not on low eGFR (*P* for interation = 0.464), which indicated that the magnitude of these associations did not differ by renal function (*P*_interaction_ > 0.1). It was worth noting that there was higher 25(OH)D level in patients with low eGFR than those without low eGFR. We speculated that vitamin D supplementation was more common among population with low eGFR for serious disorders of calcium and phosphorus metabolism, which was contributed to this inconsistent result. Additionally, no significant difference was found in 25(OH)D level across increasing DII tertiles in the population with CKD stage 4 (*P* for trend = 0.464) and CKD stage 5 (*P* for trend = 0.82). Small sample may be contributed to these results (162 patients for CKD stage 4; 83 for CKD stage 5).

Some limitations of this study should be considered. Firstly, a causal inference on the relationship between DII and 25(OH)D in CKD patients was limited because of a cross-sectional nature of this study. Second, the DII score was calculated using 24-h recall data instead of long-term dietary exposure, leading to some nutrients that have effects on 25(OH)D excluded. Thirdly, We calculated DII based on 26 dietary items and data regarding 19 other dietary items were not available in this study. Fourthly, small sample size of patients with CKD stage 4 and 5 was used to analysis, although most of the participants with CKD were in stage 1–3. It may affect the accuracy. In addition, some potential confounders, such as drug use, hemodialysis condition, were not available in NHANES data, which may influence this association. Another limitation was that 25(OH)D was only assayed at a single time point, and no repeat measurements of 25(OH)D were conducted. At last, the serum level of 25(OH)D may not accurately reflects the serum level of active form 1,25(OH)2D, which was not available in the database.

The strength of our study was that we performed subgroup analysis stratified by different setting and found similar association, suggesting that this negative association could be appropriate for different population settings. Importantly, our study was based on large sample size with more representative.

## Conclusion

Our finding demonstrated an inverse relationship of DII on 25(OH)D in patients with CKD, indicating that higher consumption of pro-inflammatory diet correlates negatively with the 25(OH)D level. This relationship was remained both in the patients with low eGFR and without low eGFR. Our research shows that anti-inflammatory diet management may reduce the reduction of vitamin D in CKD patients. Futhermore, longitudinal studies on a large sample size are needed to elucidate this relationship in CKD patients.

## Data Availability

The datasets used and analyzed during the study are available from the corresponding author on reasonable request.
